# Piezo1‐mediated M2 macrophage mechanotransduction enhances bone formation through secretion and activation of transforming growth factor‐β1

**DOI:** 10.1111/cpr.13440

**Published:** 2023-03-07

**Authors:** Guanhui Cai, Yahui Lu, Weijie Zhong, Ting Wang, Yingyi Li, Xiaolei Ruan, Hongyu Chen, Lian Sun, Zhaolan Guan, Gen Li, Hengwei Zhang, Wen Sun, Minglong Chen, Wei‐Bing Zhang, Hua Wang

**Affiliations:** ^1^ Department of Orthodontics The Affiliated Stomatological Hospital of Nanjing Medical University Nanjing China; ^2^ Jiangsu Key Laboratory of Oral Diseases Nanjing Medical University Nanjing China; ^3^ Department of Stomatology Dushu Lake Hospital Affiliated to Soochow University Soochow China; ^4^ Department of Stomatology Medical Center of Soochow University Soochow China; ^5^ Department of Pathology and Laboratory Medicine University of Rochester Medical Center Rochester New York USA; ^6^ Department of Cardiology The First Affiliated Hospital of Nanjing Medical University Nanjing China; ^7^ Department of Orthodontics Jiangsu Province Engineering Research Center of Stomatological Translational Medicine Nanjing China

## Abstract

Macrophages are multifunctional immune system cells that are essential for the mechanical stimulation‐induced control of metabolism. Piezo1 is a non‐selective calcium channel expressed in multifarious tissues to convey mechanical signals. Here, a cellular model of tension was used to study the effect of mechanical stretch on the phenotypic transformation of macrophages and its mechanism. An indirect co‐culture system was used to explore the effect of macrophage activation on bone marrow mesenchymal stem cells (BMSCs), and a treadmill running model was used to validate the mechanism in vivo for in vitro studies. p53 was acetylated and deacetylated by macrophages as a result of mechanical strain being detected by Piezo1. This process is able to polarize macrophages towards M2 and secretes transforming growth factor‐beta (TGF‐β1), which subsequently stimulates BMSCs migration, proliferation and osteogenic differentiation. Knockdown of Piezo1 inhibits the conversion of macrophages to the reparative phenotype, thereby affecting bone remodelling. Blockade of TGF‐β I, II receptors and Piezo1 significantly reduced exercise‐increased bone mass in mice. In conclusion, we showed that mechanical tension causes calcium influx, p53 deacetylation, macrophage polarization towards M2 and TGF‐β1 release through Piezo1. These events support BMSC osteogenesis.

## Graphical abstract

Piezo1‐mediated mechanical tension stimulates macrophage polarization and secretion of transforming growth factor‐beta1, promoting osteogenic differentiation. Acetylation and deacetylation of p53 play a major role in this process.

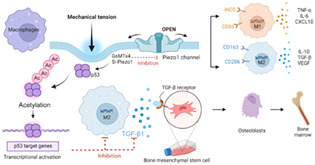

## 1 INTRODUCTION

Bone is a dynamic tissue that remodels itself constantly through two well‐coordinated processes: bone formation and resorption.^1^ For the advancement of bone regenerative medicine, it is thought to be crucial to understand the cellular and molecular mechanisms of bone regeneration. Although forces can convey parameters to cells engaged in immune‐related activities, immune cells are under stress and load during bone rebuilding, and their role in immunity has not been fully analysed.^2^, ^3^ Macrophages are multipurpose immune system cells that are essential for metabolic control, pathogen defence and tissue repair. The ability of macrophages to polarize into several functional phenotypes, emit a variety of cytokines and react dynamically to stimuli in the microenvironment leads to the diversity of macrophage roles.^4^, ^5^, ^6^ The production of tumour necrosis factor (TNF), interleukin 6 (IL‐6) and inducible nitric oxide synthase (iNOS) by M1 macrophages, as well as arginase‐1 (Arg‐1), interleukin‐10 (IL‐10), recombinant mannose receptor C type 1 (MRC1), CD206 and TGF‐β by M2 macrophages, is crucial to the process of bone reconstruction.^7^, ^8^, ^9^

Piezo1, a non‐selective Ca^2+^‐permeable cation channel, has been demonstrated to convey mechanical signals to macrophages, allowing the detection of mechanical stresses. Piezo1 is found in a variety of tissues, including macrophages.^10^ In vitro mechanical stimulation of macrophages and monocytes results in the development of a powerful and focused chemokine and pro‐inflammatory gene programme.^11^ Previous hypotheses suggested that the Piezo1 serves as a sensor of the pro‐inflammatory response brought on by stress to the myeloid cell cycle. Piezo1 regulates each component of this inflammatory mechanosensory response.^12^ According to the most recent studies, Piezo1 is downregulated in callus, which slows fracture healing, whereas its specific agonist activates Piezo1, which speeds up fracture healing and the conversion of cartilage to bone.^13^ Similar to this, He et al.'s investigations revealed that Piezo1 plays a role in the development of hypertrophic scar (HS), and intradermal injection of GsMTx4, a peptide that blocks Piezo1, shielded rats from the development of HS when it was caused by stretching.^14^

Previous research has shown that stretching can influence macrophage function and polarization towards M2. It has been demonstrated that 10% stretch intensity is optimal for activating M2‐type macrophages strongly boosting suture stem cells (SuSCs) osteogenic differentiation.^15^ Recent research has revealed that macrophages play a crucial role in bone formation by encouraging angiogenesis and osteogenesis, at least in part through secreting transforming growth factors (TGF).^16^ An in vivo investigation also indicated that TGF‐β1 is essential for bone repair.^17^ However, the mechanisms of macrophage mediation in bone development remain poorly known.

Despite numerous studies showing the osteogenic effects of macrophages on mesenchymal stem cells (MSCs), there is no agreement on which macrophage phenotype is favourable for MSC under mechanical stretch. Here, we created a cellular model of tension to examine how mechanical stretch affected the phenotypic transformation of macrophages. We revealed that the optimal mechanical stretch exhibited strong immunomodulatory effects and encouraged M2 macrophage polarization, the latter of which was a key regulator driving bone marrow mesenchymal stem cells' (BMSCs) osteogenic differentiation in an indirect co‐culture system. Mechanistically, we identified Piezo1 ion channels as tensile stress sensors in macrophages. Piezo1 triggered inflammatory responses, activated Ca^2+^, and controlled the acetylation or deacetylation of p53 in response to mechanical stress, which caused macrophages to switch from a pro‐inflammatory to an anti‐inflammatory phenotype. M2‐type macrophages produced TGF‐β1, which induced BMSC migration, proliferation and osteogenic differentiation. Running mice were given TGF‐β receptor inhibitors or Piezo1 inhibitors, which decreased the increase in bone mass brought on by exercise. In summary, we found that Piezo1‐mediated changes in calcium inward flow, as well as p53 acetylation and deacetylation, caused macrophages to polarize towards M2, secret TGF‐β1 and promote BMSC osteogenesis.

## 2 MATERIALS AND METHODS

### 2.1 Mice and the injection

Male C57BL/6 wild‐type mice aged 6 weeks (from Nanjing Medical University Experimental Animal Center, Jiangsu, China) were divided into five groups, each with five animals: the blank control group, the simple running group, the negative control group and the remaining two groups were administered drugs that were TGF‐β receptor I, II and Piezo1 inhibitor. Each mouse was raised in a separate cage to prevent fights that might mask the benefits of exercise. We used operating circumstances from other labs as references.^18^ The exercising mice ran on a treadmill (sansbio, China) at a 5° slope for 30 min. Mice adapted to the treadmill throughout the course of the first 10 min of exercise, gradually increasing their speed up to 6 m/min. All mice continued to run at a rate of 12 m/min for the remaining 20 min. One month later, the mice were euthanized and their tibia was fixed in 4% paraformaldehyde. This study was approved by the Institutional Animal Care and Use Committee of Nanjing Medical University (Approval number 2106015) and followed the National Institute of Health (NIH) policies in the Guide for the Care and Use of Laboratory Animals (NIH Publications No.80‐23, revised 1996). We performed inhibitor injections in the mice‐running model with minor modifications.^19^ Mice were given the TGF‐β receptor I, II inhibitor (LY‐364947, Med Chem Express) intraperitoneally at a dose of 10 mg/kg body weight in a solution of 200 μL of 5% DMSO and 95% sunflower seed oil (Vehicle). GsMTx4 (Med Chem Express) was previously dissolved in ddH_2_O and then administered to mice as a 10 μL (5 μM) intraperitoneal injection.^20^ Following the mice's exercise, drugs were given once every 2 days.

### 2.2 Cell culture

Male C57BL/6 wild‐type mice aged 3–4 weeks had their femurs and tibias thoroughly separated and washed with phosphate‐buffered saline (PBS). Following the removal of the bone marrow from these bones' cavities, cells were cultured in alpha minimal essential medium (α‐MEM, Gibco, Thermo Fisher Scientific) with 10% foetal bovine serum (FBS, Gibco, Thermo Fisher Scientific) and 1% penicillin–streptomycin (Gibco, Thermo Fisher Scientific). The cells were kept alive at 37°C in a humidified incubator with 5% CO_2_. After 3 days of incubation, the culture media was changed to remove non‐adherent cells. The adhering cells were washed with PBS when the confluence reached 80%–90%, and the medium was changed every 3 days while they were being grown. The cells were then cultured once more after being passaged at a ratio of 1:3. The cells from passage 3 were employed in the subsequent studies. The murine‐derived macrophage cell line RAW264.7 was purchased from the Cells Resource Center of Shanghai Institutes for Biological Sciences, the Chinese Academy of Science. It was then cultured in Dulbecco's modified Eagle's medium (DMEM, Wisent Biotechnology, Nanjing, China) containing 10% FBS (Coring, Australia), 100 IU/mL penicillin and100μg/ml streptomycin at 37°C in a humid atmosphere with 5% CO_2_.

### 2.3 Application of mechanical stretch

To apply mechanical stimulation to macrophages, RAW264.7 cells were uniformly seeded into six‐well collagen I‐coated BioFlex culture plates with flexible silicon membrane bottoms (Flexcell International Corporation, USA). A Flexcell® FX‐5000™ Tension System (Flexcell International Corporation) was used to apply cyclic sinusoidal continuous tensile strain for 0, 1, 2, 4 and 6 h (10%, 0.5 Hz). Cells were applied this optimal mechanical stretching (10%, 0.5 Hz) throughout the in vitro series. Cells were cultivated in the same plates but were not stretched as controls.

### 2.4 RNA interference

In accordance with the manufacturer's recommendations, RAW 264.7 cells were transiently transfected in six‐well plates with Piezo1 siRNA at a final concentration of 100 nM using Opti‐MEM (Gibco) supplemented with Lipofectamine 2000 Reagent (Invitrogen). We refer to the sequences of other subject groups^20^: Piezo1‐siRNA, 5′‐AGGAAGAAGCCAGAAGCUAATT‐3′ and 5′‐UUAGCUUCUGGCUCUUCCUTT‐3′, negative control (NC)‐siRNA, 5′‐UUCUCCGAACGUGUCACGUTT‐3′ and 5′‐ACGUGACACGUUCGGAGAATT‐3′.

### 2.5 Quantitative real‐time PCR

Using Trizol reagent (Invitrogen, USA), total RNA from RAW264.7 cells was extracted for quantitative real‐time PCR analysis. HiScript III Q RT SuperMix was used to reverse‐transcribe RNA (Vazyme). Next, qPCR was performed in ABI QuantStudio7 (Applied Biosystems) using ChamQ Universal SYBR qPCR Master Mix (Vazyme). The relative gene expression was quantified using the 2^−ΔΔCt^ method. The messenger RNA (mRNA) expression levels were normalized with β‐actin. Table S2 displays the qRT‐PCR primers.

### 2.6 Western blot analysis

Total proteins from macrophages were extracted using RIPA lysis buffer. The protein concentration of the cell lysates was determined using a BCA protein assay (Beyotime, China) and separated using 10% sodium dodecyl sulphate‐polyacrylamide gel electrophoresis (SDS‐PAGE) before being transferred to a polyvinylidene fluoride membrane. After blocking with 5% skimmed milk or BSA blocking solution in Tris‐buffered saline containing 0.05% Tween 20 (TBST), the membranes were incubated overnight with the following primary antibodies: anti‐Runx2, anti‐OSX, anti‐OPN, anti‐Col1, anti‐iNOS, anti‐Arg‐1, anti‐p53, anti‐ac‐p53, anti‐β‐actin and anti‐GAPDH. After primary antibody incubation, membranes were washed, incubated with a secondary anti‐rabbit or anti‐mouse antibody (Beyotime, China, 1:8000) for 1 h at room temperature and then visualized using ECL detection kits (Beyotime, China). The dilutions and suppliers of antibodies used in this study are listed in Table S1.

### 2.7 Flow cytometry

Flow cytometry was used to analyse the expression of the M1 marker CD86 and the M2 marker CD206 in order to describe the M1 and M2 macrophage phenotypes. 0.25% trypsin in ethylenediaminetetraacetic acid was used to separate the cells, and they were then thrice washed with PBS. The cells were incubated in PBS with APC‐F4/80, PE‐CD86 antibody and FITC‐CD206 antibody for 60 min at 4°C in the dark. The labelled cells were measured using a flow cytometer (Accuri C6, BD Biosciences, USA). The negative control cells were those with no additional antibodies.

### 2.8 Cell immunofluorescence

Upon the cell density reached roughly 70%, we washed the cells three times in PBS before fixing them for 30 min in 4% paraformaldehyde. We used goat serum (BOSTER, AR009) to block the samples for 30 min at room temperature. After that, we chose the primary antibodies against F4/80, CD206, Ac‐p53 and Piezo1 for overnight incubation at 4°C. On the second day, the samples were washed in PBS three times. Subsequently, the species‐matched secondary antibodies (SA00009‐2, Proteintech) were applied, and the nucleus was stained with 4′,6‐diamidino‐2‐phenylindole as a counterstain (DAPI, VECTASHIELD, H‐1500). Cells were similarly fixed in 4% paraformaldehyde for 15 min and then soaked in 0.5% Triton‐X for 5 min prior to phalloidin staining. According to the manufacturer's instructions, DAPI staining and phalloidin staining (Cytoskeleton, Cat# PHDR1) were carried out. For 5 or 30 min, cells were fixed in DAPI and phalloidin staining solution. The images were captured using an immunofluorescence microscope (Olympus, Japan).

### 2.9 Transwell assay

Cell migration was performed in 6.5 mm Transwell® with 8.0 μm Pore Polycarbonate MembraneInserts (Corning, NY, USA). The system's lower chamber contained 600 μL conditioned medium while the upper chamber contained 200 μL of BMSCs suspended in serum‐free medium. The transwell filter system containing the BMSCs was subsequently placed in a humidity incubator. After 12 h of incubation at 37°C, BMSCs were fixed with 4% paraformaldehyde, and the non‐traversed cells that remained on the filter's upper surface were carefully removed with a cotton swab. Migrated (traversed) cells on the lower side of the filter were stained with crystal violet for 30 min, and then counted in five randomly selected fields under an inverted microscope (Olympus, Tokyo, Japan).

### 2.10 CCK8 assay

For the CCK8 proliferation assay, BMSCs (3000 cells per well) were seeded in 96‐well plates and incubated at 37°C with 5% CO_2_. The plate was subsequently processed with Cell Counting Kit 8 (CCK8) solution (10 μL; Dojindo, Japan) for specified durations (0, 12, 24, 36 and 48 h) at 37°C. Optical density was measured at 450 nm to calculate the percentage of cell viability on a microplate reader (Spectramax, USA).

### 2.11 5‐ethynyl‐2′‐deoxyuridine incorporation assay

The proliferation of 5 × 10^4^ BMSCs was evaluated using 5‐ethynyl‐2′‐deoxyuridine (EdU) incorporation assay reagent (RiboBio, China) in a 96‐well plate. Simply put, the cells were exposed to the EdU reagent for 4 h once cell growth in the plate reached 80% confluence. Cells were fixed, permeabilized and dyed with 4′,6‐diamidino‐2‐phenylindole (DAPI) solution before being observed under a fluorescent microscope (Leica).

### 2.12 Scratch wound‐healing assay

For the scratch wound‐healing assay, cells were seeded at a density of 1 × 10^6^ cells/well (in six‐well plates), and a scratch was formed on the cell monolayer 6 h later. The cells were placed in a humidified incubator with 5% CO_2_ at 37°C after being washed three times with serum‐free media. They were then monitored for a further 24 h. New scratch width/original scratch width was used as the unit of measurement for cell migration.

### 2.13 Alkaline phosphatase staining

The cells were PBS‐washed before being fixed in 4% paraformaldehyde at room temperature for 30 min. Following that, the samples were stained with the BCIP/NBT Alkaline Phosphatase Color Development Kit (Beyotime, China) according to the manufacturer's instructions. The images were captured with a scanner (GE Image Scanner III). The staining was simultaneously viewed under a microscope. We measured the alp enzyme in three randomly chosen fields using Image‐J software.

### 2.14 Osteogenic differentiation alizarin red staining

BMSCs were cultured in plates with different conditional mediums for 21 days. Cells were rinsed three times in PBS after a 21‐day osteogenic induction, and then paraformaldehyde was used to fix the cells for 30 min at room temperature. The samples were then washed and stained at room temperature with Alizarin Red S (Leagene, China). The images were captured with a scanner (GE Image Scanner III). The staining was simultaneously viewed under a microscope, and after that, Hexadecylpyridinium chloride monohydrate (H108696, Aladdin) was employed for quantification.

### 2.15 Ca2+ fluorescence image

RAW264.7 cells were cultured in BioFlex culture plates after Piezo1 silence or Yoda1 injection. Cells were then loaded with Fluo‐3 AM (5 μM; Beyotime, China) and simultaneously treated to mechanical stretch (10%, 0.5 Hz) for 0–6 h. The macrophages were washed with PBS three times after mechanical stretch. Ca^2+^ fluorescence images were captured with a fluorescent microscope (Leica).

### 2.16 Enzyme‐linked immunosorbent assay

TGF‐β1 expression levels were quantified for inflammatory cytokine measurement. The supernatant was utilized to identify macrophages after they had been stretched. ELISA was performed as directed by the manufacturer's instructions (R&D Systems, American).

### 2.17 μCT analysis

Tibia bones were extracted, fixed in 4% paraformaldehyde solution and scanned with a high‐resolution micro‐ct (Skyscan 1176, Bruker, Germany) at a resolution of 15.6 μm. Three‐dimensional images were reconstructed for analysis (CTAn, Skyscan), and cross‐sectional images of the distal tibia were employed. Based on the selection of the volume of interest (VOI), which was generated in accordance with the previous report,^21^ volumetric measurements were performed. The proximal metaphysis was selected as a 2‐mm region of interest, beginning at the distal end of the proximal growth plate and extending distally. Based on that, it was set in at 50 to 255 to calculate the bone volume fraction (BV/TV), trabecular thickness (Tb.Th), number (Tb.N) and separation (Tb.Sp).

### 2.18 Histology and immunohistochemical staining

After being fixed in paraformaldehyde overnight, the samples were decalcified in 14% EDTA and embedded in paraffin. The tibias were cut into 4‐μm‐thick sections. The slices were stained with haematoxylin and eosin (H&E). The trabecular structure and osteocyte lacunae were examined to assess bone alterations. Femurs were first deparaffinized and heated to facilitate antigen retrieval for immunohistochemical tests. Goat serum was used to block tissue slices for 30 min at room temperature. TGF‐β1 and F4/80 primary antibodies were added and incubated overnight at 4°C.The sections were then treated with fluorescein Cy3‐conjugated secondary anti‐body (Beyotime) and fluorescein CoraLite488‐conjugated secondary antibody (Proteintech) on the second day. Nuclei were stained with DAPI. A fluorescent microscope was used to obtain the images (Carl Zeiss).

### 2.19 Statistical analysis

To limit experimental error, all tests were repeated three times. The data were recorded as mean ± SD. Graphpad Prism 8.0 (GraphPad, San Diego, CA, USA) was used for statistical analysis. *p*‐Values were calculated using a two‐way analysis of variance (ANOVA) with Bonferroni's correction or multiple *t*‐tests. A *p*‐value of <0.05 was judged statistically significant.

## 3 RESULTS

### 3.1 Mechanical tension induces macrophage polarization

**FIGURE 1:**
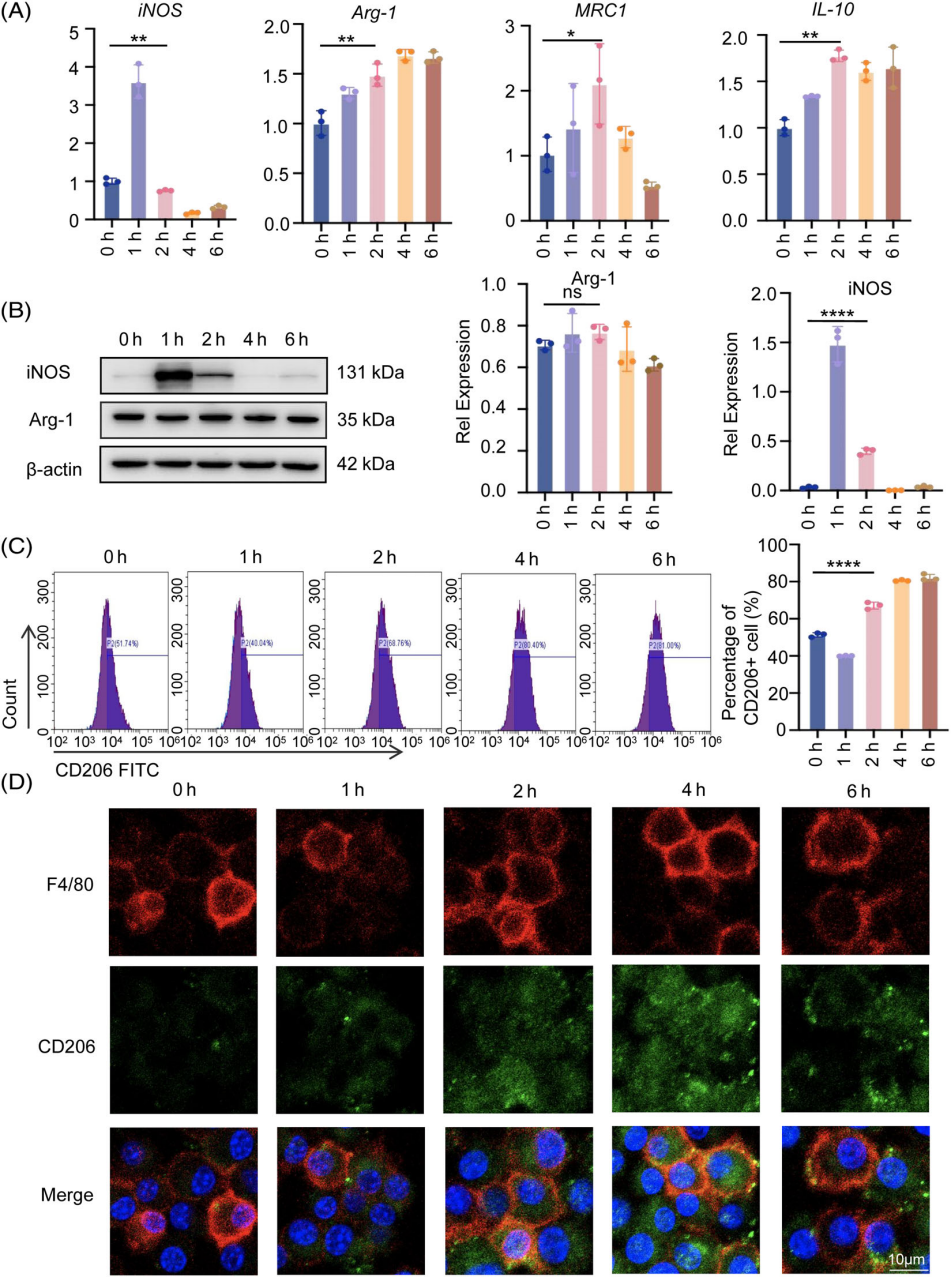
Mechanical tension induces macrophage polarization. (A) Real‐time RT‐PCR was used to assess the mRNA levels of iNOS, Arg‐1, MRC1 and IL‐10 in RAW264.7 cells as a result of the 0–6 h of tension. (B) Western blot was used to identify the protein expression of iNOS and Arg‐1 in RAW264.7 cells under stress for 0–6 h. GAPDH was utilized as the standard to quantify iNOS and Arg‐1. (C) The expression of CD206 in RAW264.7 cells under tension was detected by flow cytometry, and the statistical results were displayed. (D) M2 macrophages in RAW264.7 cells under the tension of 0–6 h were visualized using immunofluorescence staining of F4/80 (red) and CD206 (green). The cell nuclei were detected with DAPI. Scale bar: 10 μm. Data represent the mean ± SD. *p < 0.05, **p < 0.01, ***p < 0.001.

**FIGURE 2:**
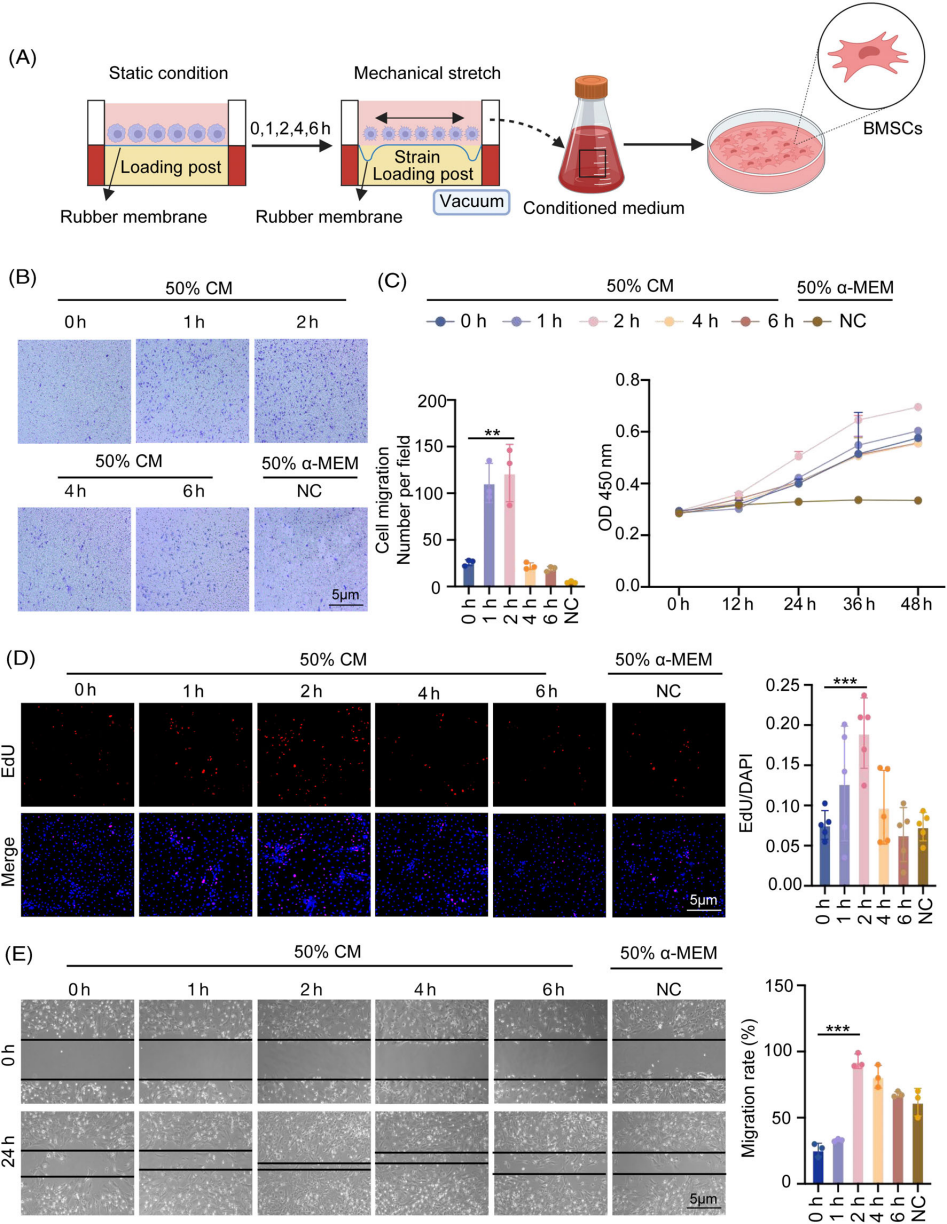
Macrophage‐conditioned medium under tension promotes the proliferation and migration of BMSCs. (A) Diagram of macrophages under tension with the Flexcell® FX‐5000™Tension System. (B) Effects of conditioned medium of macrophages under 0–6 h tension on vertical migration of BMSCs are demonstrated by ×4 micrographs of BMSCs in different groups at the bottom of the migration transwell culture chamber and a histogram of cell counts based on ×4 magnification fields. (C) Cell Counting Kit 8 (CCK8) assays were performed to explore BMSCs proliferation following treatment with the macrophage‐conditioned medium under 0–6 h of tension (magnification ×100). (D) Additionally, BMSCs proliferation was investigated using 5‐ethynyl‐2′‐deoxyuridine (EdU) tests (magnification ×100). (E) Effects of conditioned medium on lateral migration, as shown by the initial and final (24 h) micrographs of the scratch in the wound healing assay. Bar graph of final distance/initial distance rate of each group. Data represent the mean ± SD. *p < 0.05, **p < 0.01, ***p < 0.001.

Previous in vitro experiments have demonstrated that M2 macrophages can induce MSCs osteoblast differentiation.^22^ We proposed that mechanical tension would stimulate M2‐type macrophage transformation, causing BMSCs to initiate osteogenesis. RAW264.7 cells were seeded on BioFlex six‐well cell culture plates and subjected to mechanical strain for 0–6 h to examine what happened to macrophages (Figure 2A). Phalloidin was used to stain macrophages both before and after mechanical stretching (Figure S1A). As the acceleration period was prolonged, additional pseudopods on macrophages appeared, indicating the presence of more M2‐type macrophages. The gene expression levels of the three main cytokines released by M2 macrophages were evaluated using RT‐QPCR: Arg‐1, IL‐10 and MRC1. The expression of MRC1 and IL‐10 peaked at the second hour of stretching. After being subjected to mechanical stress, iNOS, the major cytokine released by M1‐type macrophages, reached a peak (almost four times that of controls) in the first hour and thereafter fell to a level below that of controls (Figure 1A). To investigate the polarization response of macrophages to tension, Western blot analysis of the fraction of M1‐ and M2‐polarized macrophages was utilized (Figure 1B). Flow cytometric analysis revealed that stress increased the proportion of CD206^+^ cells in RAW264.7 cells, further demonstrating that stress alters the phenotype of macrophages (Figure 1C). Additionally, dual immunostaining for F4/80 and CD206 (M2 markers) was performed. Notably, the increase in M2 macrophages was more pronounced in the second hour, and RAW264.7 cells strongly expressed F4/80 (Figure 1D). These results demonstrated that tension caused macrophages to M2‐like polarization in a time‐dependent manner, with the effect of tension becoming more obvious at 2 h. Therefore, 2 h was chosen as the time point for additional research.

### 3.2 Macrophage‐conditioned medium under tension promotes the proliferation and migration of BMSCs

To examine if the macrophage‐derived medium has any effect on BMSCs' migration, BMSCs and macrophages were indirectly co‐cultured (Figure 2A). The purpose of the transwell migration test was to examine the impact of the conditioned media on the vertical migration of BMSCs. We placed 600 μL of conditioned media in the lower compartment and 200 μL of BMSCs cell suspension in the upper chamber. After 12 h, the results revealed that 2‐h tension had the greatest potential to migrate, with significant differences from the control group (Figure 2B). Additionally, the proliferation of BMSCs in indirect co‐cultured with RAW264.7 cells was impacted. The Cell Counting Kit 8 (CCK8) and 5‐ethynyl‐2′‐deoxyuridine (EdU) assays were performed after adding the conditioned medium. BMSCs in the various treatment groups (0, 1, 2, 4 and 6 h of tension) all showed significantly higher proliferative potential, with the highest result occurring at 2 h of tension (Figure 2CD). The scratch wound healing assay was used to examine the lateral migratory capabilities of BMSCs. Cells receiving conditioned media showed greater capacity for lateral migration at the second hour than control BMSCs (Figure 2E). Considering this, conditioned medium from stressed RAW 264.7 cells dramatically increased the migration of BMSCs, indicating its likely positive effects on tissue healing via enhancing stem cell homing.

### 3.3 Macrophage‐derived medium promotes osteogenic differentiation of BMSCs

**FIGURE 3:**
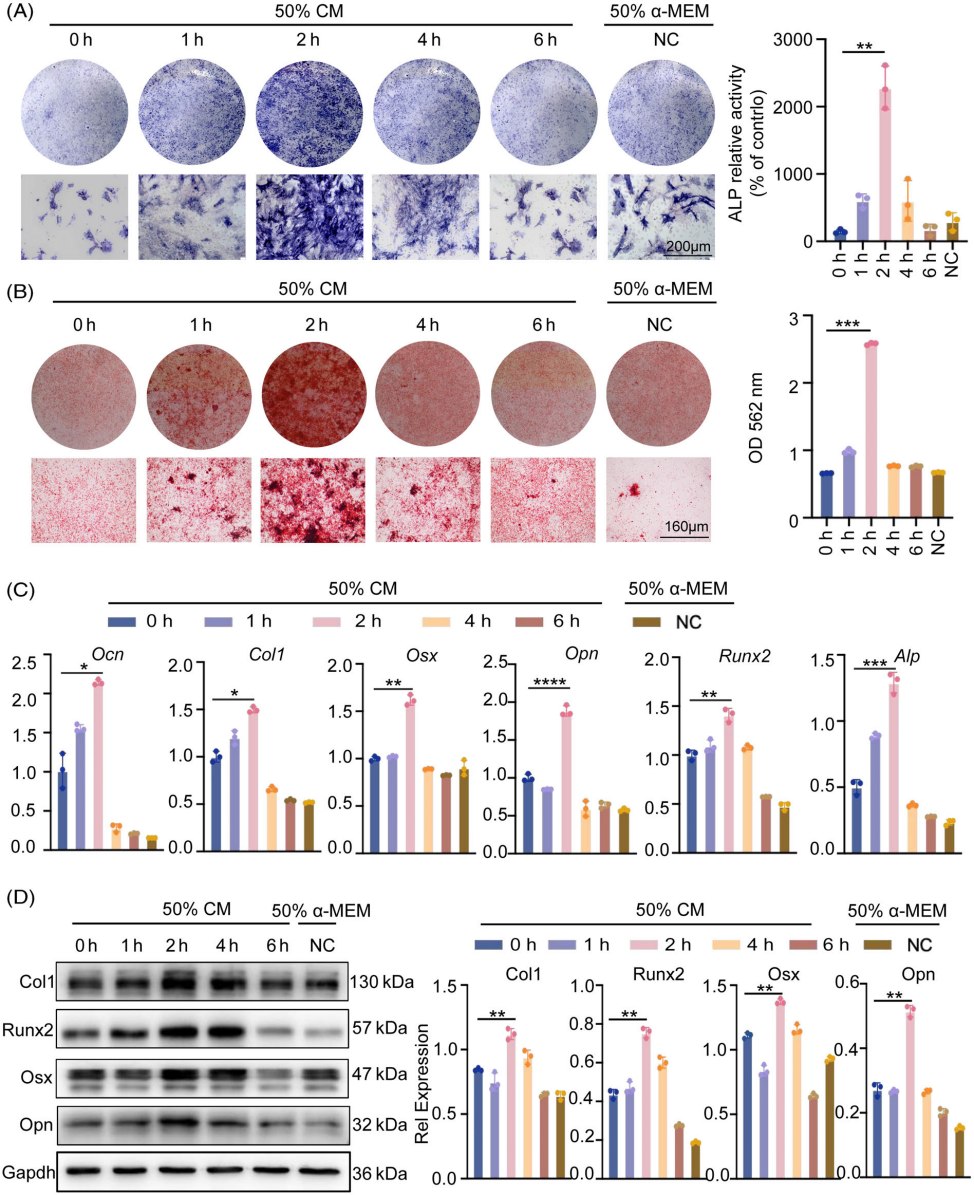
Macrophage‐derived medium promotes osteogenic differentiation of BMSC. (A) In osteogenic differentiation medium, BMSCs treated with macrophage supernatants were induced to undergo osteogenesis. ALP expression in BMSCs was measured by the ALP staining method. Gross scanning images are shown at the top (scale bar: 1 mm), expanded images are shown at the bottom (magnification: ×250, scale bar: 160 μm), and a quantification of the gross scanning images is shown on the right. (B) The osteogenic differentiation of BMSCs was evaluated by Alizarin Red staining after 21 days. (C) Ocn, Col1, Osx, Opn, Runx2 and Alp in BMSCs were measured by real‐time RT‐PCR. GAPDH was used for normalization. (D) The protein expression of Col1, Runx2, Osx and Opn in BMSCs with conditioned medium were detected by western blot. GAPDH was used for normalization. Data represent the mean ± SD. *p < 0.05, **p < 0.01, ***p < 0.001.

To study the effects of the macrophage immune response on osteogenic differentiation, BMSCs were stimulated to differentiate into osteoblasts in their respective conditioned media with varying periods of tension. As a result of our early research, we chose a 50% conditioned medium concentration (Figure S2A,B). Alkaline phosphatase and alizarin red S were stained after 7 and 14 days of continuous culture. There was no discernible difference in osteogenic staining between the control group and the 0‐h group. Notably, there was a significant increase in the BMSCs' osteogenic potential in the 2 h stretching group, but not between the subsequent stretching group and the control group. This might be as a result of cytokines associated to macrophages released in the supernatant (Figure 3A,B). Mineralization in the 2‐h group was substantially higher than that in the control group (about two times). Consistent with the mineralization degree, osteogenic mRNA and protein expression levels of Col1, Runx2, Osx and Opn were considerably up‐regulated in the conditioned media of 2‐h stretching‐stimulated BMSCs (Figure 3C,D). According to the aforementioned findings, 2 h of stretching may promote osteogenesis via controlling M2 macrophage polarization in an immunological milieu.

### 3.4 Piezo1 is required for the mechanically induced M2 polarization

**FIGURE 4:**
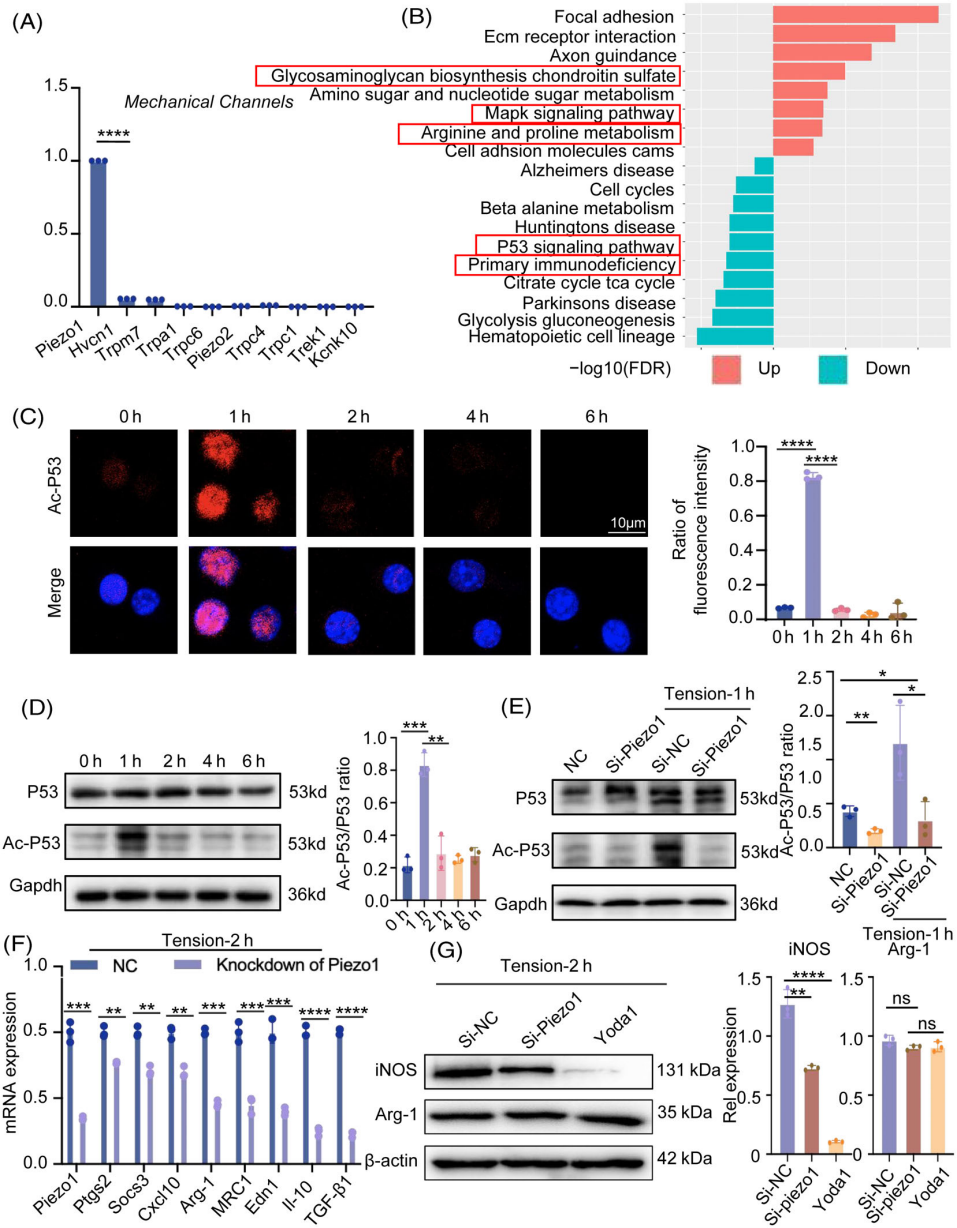
Piezo1 is required for the mechanically induced M2 polarization. (A) mRNA levels of calcium channels in RAW264.7 cells were determined by RT‐PCR. (B) Differential signalling pathways were analysed from the database (GSE139121). (C) Immunofluorescent staining of acetyl‐p53 (red) in RAW264.7 cells under the tension of 0–6 h. Blue indicates DAPI staining of nuclei. Scale bar: 10 μm. (D) Western blot was used to identify the p53 and acetyl‐p53 protein expression in RAW264.7 cells as a result of 0–6 h of tension. GAPDH was used for normalization. (E) In the control or Piezo1 knock‐down groups, p53 and acetyl‐p53 protein expression were tested under static or tension conditions. (F) Piezo1, Ptgs2, Socs3, Cxcl10, Arg‐1, MRC1, Edn1, IL‐10 and TGF‐**β**1 were detected by qPCR in control or Piezo1 knock‐down groups that had been cultivated under tension for 2 h. (G) Western blotting was used to identify the protein expression of iNOS and Arg‐1 in three groups (the control group, the Piezo1‐knockdown group and the Yoda1‐administered group) during 2 h of tension. Data represent the mean ± SD. *p < 0.05, ** *p* < 0.01, ***p < 0.001.

To determine why applying stress would impact macrophages polarization, we detected the calcium ion signal. The fluorescence intensity of calcium ions varied with the time of applied tension, and there was a considerable influx of calcium ions at 2 h (Figure S3A). We then examined at calcium‐associated ion channels in macrophages and found that Piezo1 expression was extremely high in comparison to other channels (Figure 4A). We therefore hypothesized that the tension‐induced polarization of macrophages was closely connected to the calcium inward influx mediated by Piezo1. Piezo1 was dramatically raised after 2 h of tensioning, according to RT‐PCR and cellular immunofluorescence outcomes (Figure S3B,C). The database (GSE139121) is examined based on the Piezo1 expression level. Each group consists of three samples, and the upper and lower enrichment pathways are decided by whether the value is more than the median, indicating a high‐expression group, or lower, indicating a low‐expression group. With Piezo1 knockdown, the p53 signalling pathway was discovered (Figure 4B). Cellular immunofluorescence revealed that, in contrast to the control and other time periods, the fluorescence intensity of acetylated p53 was extraordinarily high 1 h after tension administration (Figure 4C). It was consistent with the change in iNOS after 0–6 h of after tension. Next, we looked at how p53 and acetylated p53 changed from the protein level over time. We found that p53's protein expression did not significantly change over time compared to the control, but acetylated p53 did, in accordance with our findings from immunofluorescence, show a peak after 1 h of tension. One cannot help but wonder if the acetylation of p53 and, hence, macrophage polarization may be impacted by mechanical stress (Figure 4D). We used lentiviral intervention of Piezo1 on macrophages to knock it down in order to investigate whether alterations in Piezo1 had an impact on p53 and its acetylation. Proteins were collected with and without tension from the control and si‐Piezo1 groups. Western blot analysis revealed that acetylated p53 was substantially lower in the tiny interfering group than in the Si‐NC group after 1 h of stress application (Figure 4E), indicating Piezo1 knockdown increased the expression of acetylated p53. Because acetylated p53 was consistent with the shift in iNOS, we wondered if Piezo1 deficiency may alter macrophage polarization. Proinflammatory genes such Cxcl10, Ptgs2, Socs3, Edn1 and IL‐10, as well as the M2 macrophage marker, were strongly down‐regulated in RAW264.7 cells under stress after Piezo1 was knocked down by siRNA (Figure 4F). Then, three groups—the control group, the Piezo1 knockdown group, and the Piezo1 activator group with Yoda1—were established. Western blot was used to check the polarization of macrophages after 2 h of applied stress. The findings revealed that almost all of the macrophages in the Piezo1 activator group were M2 type, and that iNOS expression in the Piezo1 knockdown group was almost half that in the control group (Figure 4G). As a result, it is believed that Piezo1 is essential for macrophages to transition to the M2 phenotype.

### 3.5 Piezo1 knockdown hampers the proliferation, migration and osteogenic differentiation of BMSCs

**FIGURE 5:**
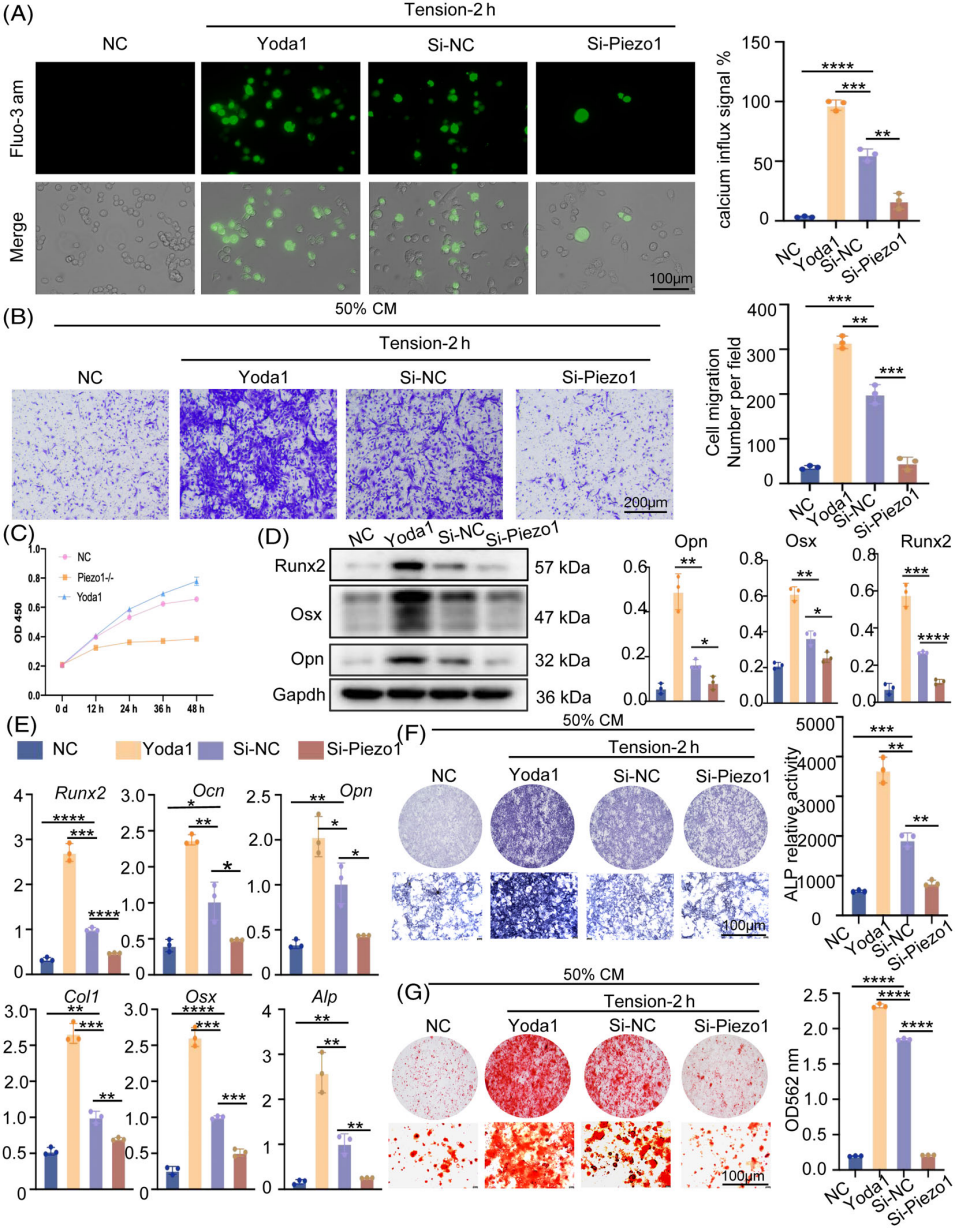
Piezo1 knockdown hampers the proliferation, migration and osteogenic differentiation of BMSCs. (A) Fluorescence intensity of Ca^2+^ after Piezo1 knockdown or treatment with 1 μmol/L Yoda1 as measured by the Fluo‐3 AM probe under a fluorescence microscope. (B) Effects of 1 μmol/L Yoda1 or knockdown of Piezo1 in macrophages on vertical migration of BMSCs under static 2‐h tension, as shown by ×4 micrographs of BMSCs in different groups at the bottom of the migration transwell culture chamber. (C) Cell Counting Kit 8 (CCK8) was performed to explore BMSCs proliferation following treatment with 1 μmol/L Yoda1 or knockdown of Piezo1. (D) The protein expression of Runx2, Osx and Opn in BMSCs were detected by western blot. GAPDH was used for normalization. (E) Col1, Osx, Runx2, Ocn, Opn and Alp in BMSCs were measured by real‐time RT‐PCR. GAPDH was used for normalization. (F) ALP expression in BMSCs was measured by the ALP staining method. The top are gross scanning images (scale bar: 1 mm), and the lower are enlarged images (magnification: ×250, scale bar: 160 μm). (G) After 21 days, the osteogenic differentiation of BMSCs was evaluated by Alizarin Red staining. The top are gross scanning images (scale bar: 1 mm), and the lower are enlarged images (magnification: ×250, scale bar: 160 μm). Data represent the mean ± SD. *p < 0.05, **p < 0.01, ***p < 0.001.

Interestingly, Piezo1 was knocked down by siRNA in RAW264.7 cells, which resulted in a decrease in calcium influx as measured by Fluo‐3 AM. Yoda1 has previously been identified as a specific agonist of Piezo1, and PCR results showed that 1 μL/mL of Yoda1 maximally agonized Piezo1 in a manner that was five times stronger than the control (Figure S4A). A significant increase in the inward flow of calcium ions was observed in macrophages after adding of 1 μL/mL of yoda1 to the medium and 2 h of mechanical tension (Figure 5A). Moreover, the migration and proliferation of BMSCs matched the results of calcium inward flow. Yoda1 administration showed the greatest effect on BMSCs migration after 2 h of strain. After Piezo1 knockdown, the conditioned medium of macrophages exhibited little effect on BMSCs, about the same as the control group under static conditions (Figure 5B,C). The osteogenic differentiation capacity of BMSCs was further examined using PCR, western blot, alkaline phosphatase and alizarin red staining. Under the same conditions, after Piezo1 overexpression, the supernatant of macrophages greatly increased the osteogenic capacity of BMSCs. Contrarily, following 2 h of tension exposure for macrophages, knockdown of Piezo1 had little impact on BMSCs in conditioned media (Figure 5D–F). Overall, Piezo1 is critical for the transmission of mechanical forces and even regulates whether mechanical tension can impact macrophage polarization, which would then affect BMSC migration, proliferation and osteogenic differentiation.

### 3.6 M2 macrophages promote the osteogenic differentiation of BMSCs by secreting TGF‐β1

**FIGURE 6:**
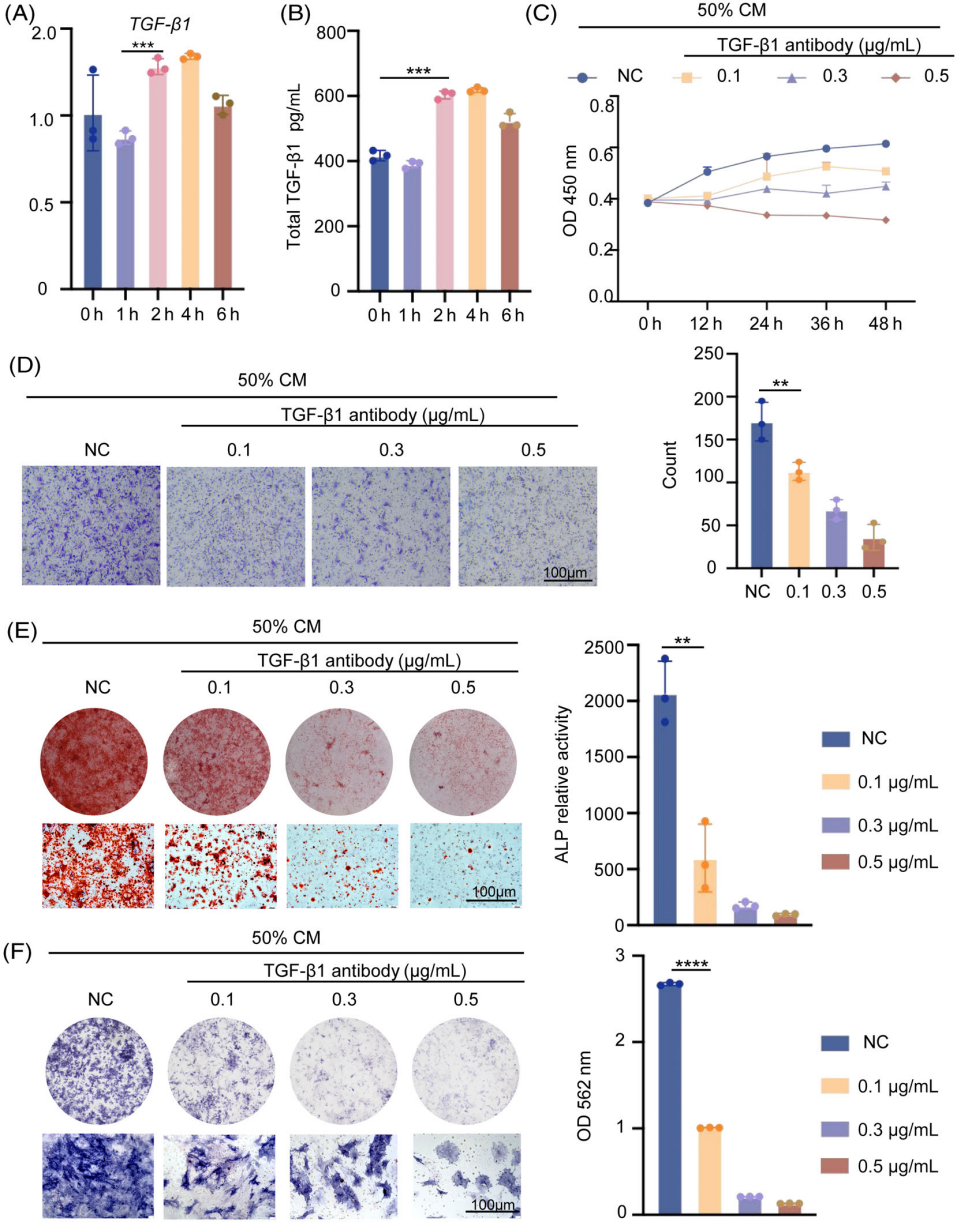
M2 macrophages promote the osteogenic differentiation of BMSCs by secreting TGF‐β1. (A) TGF‐β1 in RAW264.7 cells was measured by real‐time RT‐PCR. GAPDH was used for normalization. (B) TGF‐β1 secretion from macrophages under various stress situations is measured using an ELISA. (C) Cell Counting Kit 8 (CCK8) was used to investigate BMSCs proliferation after treatment with various concentrations of TGF‐β1 antibody. (D) TGF‐β1 antibody effects on BMSC vertical migration, as indicated by four micrographs of BMSCs in separate groups at the bottom of the migration transwell culture chamber. (E) BMSCs treated with TGF‐β1 antibody were stimulated into osteogenesis in osteogenic differentiation medium. The ALP staining approach was used in BMSCs. The top photos are gross scanning images (scale bar: 160 μm), whereas the bottom images are expanded images (magnification: 250, scale bar: 160 μm). (F) The osteogenic differentiation of BMSCs was assessed by Alizarin Red staining after 21 days. The photos on top are gross scanning images (scale bar: 160 mm), while the images on the bottom are expanded images (magnification: ×250, scale bar: 160 μm). Data represent the mean ± SD. *p < 0.05, **p < 0.01, ***p < 0.001.

We have already shown that stress causes macrophages to polarize towards M2 through Piezo1, which impacts BMSCs migration, proliferation and osteogenesis. TGF‐β1 and other cytokines that have an impact on BMSC osteogenesis are produced after macrophage polarization.^23^ To evaluate the expression of macrophage‐secreted TGF‐β1 under tension, the mRNA level of TGF‐β1 in RAW264.7 cells was measured using real‐time RT‐PCR, and protein level of TGF‐β1 in supernatants was measured using ELISA. The results showed that mechanical tension could stimulate TGF‐β1 secretion, especially at 2 h (Figure 6A,B). BMSCs in the various treatment groups all showed considerably decreased proliferative potential in the CCK8 assay, which was conducted 12–48 h after adding varying doses of TGF‐β1 antibody to the indirect co‐culture system (Figure 6C). The transwell migration assay was used to assess the migration capacities of BMSCs treated with antibodies, which showed the similar trend as proliferation. Quantification showed that BMSCs treated with 0.5ug/ml TGF‐β1 antibody had the lowest migratory capacity, with significant differences compared to the control group (Figure 6D). The effects of osteogenesis in the indirect co‐culture system were investigated using ALP and Alizarin Red staining. The findings demonstrated that TGF‐β1 antibody therapy significantly decreased BMSCs' expression of osteogenesis (Figure 6E,F). Therefore, we draw the conclusion that mechanical tension causes macrophages to polarize towards M2 and release TGF‐β1, which in turn promotes BMSCs migration, proliferation and osteogenic differentiation.

### 3.7 Mice treated with the TGF‐β receptor I, II inhibitor or Piezo1 inhibitor show poor bone remodelling

**FIGURE 7:**
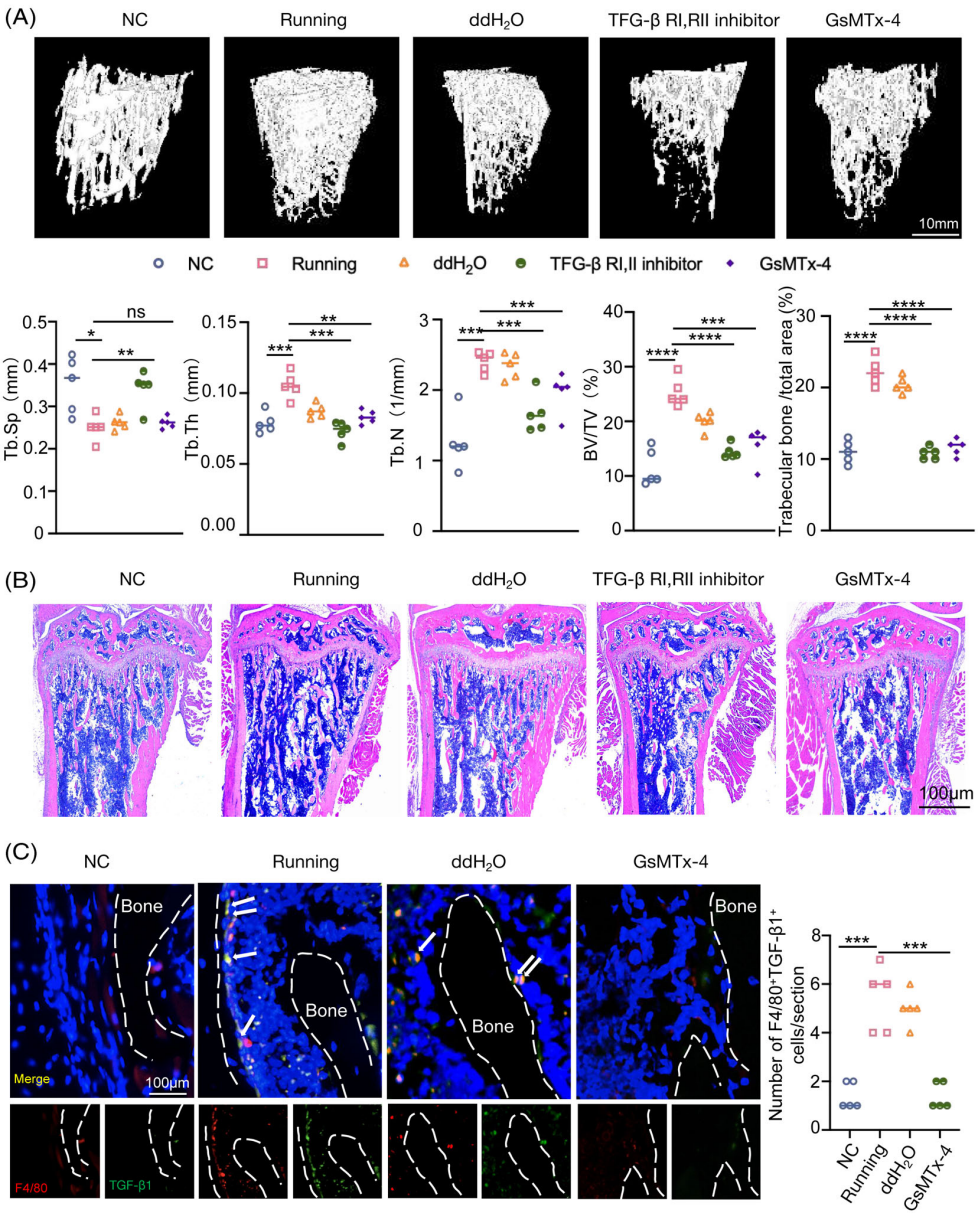
Knockdown of TGF‐β1 and Piezo1 both reduced bone remodelling in running mice. (A) μ‐CT images of the distal tibia trabecular bones isolated from 10‐week‐old male WT‐mice and mice that had been treated with TGF‐β receptor I (RI), receptor II (RII) inhibitor and Piezo1 inhibitor. μ‐CT analysis of interesting region for bone volume per tissue volume (BV/TV), trabecular thickness (Tb.Th), trabecular number (Tb.N), trabecular spacing (Tb.Sp). (B) Representative HE‐staining images of the tibial metaphysis after 1 month of exercise. (C) Representative immunofluorescence staining and quantification of F4/80^+^ (red) TGF‐β1^+^ (green) cells on the distal tibia. Scale bar, 100 μm. White arrows indicated F4/80^+^ and TGF‐β1^+^ cells.

Bone formation is a complex process involving interactions between the skeleton and the immune system. A running model was developed to investigate the roles of Piezo1 and TGF‐β1 during bone regeneration. None of the mice in the control group ran. The remaining mice exercised for 30 min each day, 5 days a week, for 4 weeks. The first 10 min were spent running at 6 m/min with an acceleration of 1 m/s^2^, while the remaining 20 min were spent running at 12 m/min. Water, Piezo1 inhibitor (GsMTx‐4), or TGF‐β receptor I, II inhibitor (LY‐364947) were injected. Running significantly increased bone mass in mice, but we could see that mechanical force transmission was also stopped when Piezo1 was inhibited, and bone mass in the GsMTx‐4‐injected group was almost the same as in the control group (i.e., no running group). TGF‐β1 released by macrophages failed to play a function after TGF‐β receptor I, II was blocked, resulting in severe bone repair in the tibia of mice (Figure 7A). The HE staining results showed that the number of bone trabeculae in the running group was significantly higher. However, both the non‐running group and the group that received a medication injection had significantly fewer bone trabeculae (Figure 7B). Immunofluorescence staining revealed that running greatly raised TGF‐β1 secretion by F4/80^+^ positive macrophages, but Piezo1 inhibitors significantly inhibited this effect (Figure 7C). Therefore, Piezo1 inhibition drastically decreased TGF‐β1 secretion from macrophages, thereby reducing the in vivo increase in bone mass caused by exercise.

## 4 DISCUSSION

Mechanical forces are essential for maintaining skeletal growth and homeostasis. Bone can modify its structure in response to mechanical stimuli, resulting in the formation of new bone at the site of applied force via osteoclastic and osteoblastic activities.^24^ On the other hand, extended periods of inactivity without physical activity or extended time spent in a microgravity environment in space result in a rapid loss of bone mass and bone strength.^25^, ^26^ Many previous studies have shown that macrophages interact with the skeletal system and play a key role in bone immunity. Piezo1 is an important regulator of macrophage phagocytosis.^27^ Macrophage polarization caused by mechanical stimulation has an impact on stem cells, which in turn impacts bone repair.^28^ It has been demonstrated that M2 macrophages aid in the differentiation of MSCs differentiate into osteoblasts.^22^, ^29^, ^30^ According to one view, a brief inflammatory phase is required for increased bone production.^31^ The most likely cause of this discrepancy is a variety in study design. It might be as a result of the multiple macrophage sources and co‐culture concentrations, for example.^32^

Although we all know that M2 macrophages release TGF‐β, the exact mechanism by which they encourage BMSCs to differentiate into osteoblasts is unknown. TGF‐β1 is described as the member of the TGF‐β family proteins that has undergone the most investigation because of its wide range of involvement in the control of cell proliferation and differentiation, wound healing and immune system, as well as its critical roles in pathology, such as skeletal diseases, fibrosis and cancer.^33^ We successfully inhibited TGF‐β1 signalling expression by blocking the receptor of TGF‐β1, which is consistent with the results of other research groups.^34^ Exogenous TGF‐β1 is able to induce chondrocyte bone formation.^35^ It has been shown that TGF‐β1 and BMP signalling interact to control the hBMSCs' ability to differentiate genetically.^36^ Further research is needed in our follow‐up to determine the type of transcriptional reprogramming that will be generated when TGF‐β1 binds to its receptor.

According to the results of the current study, Piezo1 regulates the bone immune system in macrophages by detecting mechanical stress. Consistent with prior findings, macrophages polarize to M2 type at 10%, 0.5 Hz mechanical stretch, although previous studies did not investigate the ideal stretch time of macrophages in terms of spatiotemporal principles. By stretching macrophages at various intervals, we discovered that 2 h of tension exposure had the greatest impact on bone repair.

Additionally, the activation mechanisms of M2 macrophages in response to mechanical stretching remain unclear. By analysing the database (GSE139121), we found that the p53 axis is important in the mechanical force‐dependent Piezo1 that leads to macrophage polarization.^37^ However, since the database was created from bone tissue, it would be more accurate for us to gather RNA from macrophages and perform RNA‐seq analysis on it. p53 has already been linked to macrophage polarization in a number of studies. For instance, chemically induced p53 lowered M2‐related genes and phagocytic activity.^38^ Iron overload‐induced M1 polarization in macrophages via promoting p53 acetylation.^39^ However, there are currently very few research examining the relationship between mechanical stresses and p53 acetylation. Only studies have demonstrated that the loss of Piezo1 promotes the accumulation of p53, which causes a significant induction of senescence and, eventually, serious abnormalities in skeletal muscle regeneration.^33^ Macrophages attained their maximal M1 polarization at 1 h, and then gradually changed to become M2‐type macrophages. We have reason to believe that the mechanism at work is connected to the deacetylation of p53, but this needs to be investigated more in the further. Additionally, our research demonstrates that the Piezo1‐induced reduction of calcium inward flow can significantly speed up the acetylation of p53, converting macrophages to M1—a state that is exceedingly harmful to our osteogenesis process. These findings may provide fresh perspectives on how external mechanical forces and internal biological signalling pathways interact to eventually polarize cells, which in turn affects bones as well as other tissues and organs.

Bone consists of cortical bone and bone trabeculae.^40^ In vitro studies, in vitro experiments showed that giving macrophages the right kind of mechanical stimulation encourages MSCs osteogenesis. A study employing a model of direct tibial compressive stress loading showed that CD68^+^F4/80^+^ macrophages sense mechanical signals via Piezo1 and secrete TGF‐β, which encourages the production of periosteal bone. Recent research has also demonstrated the advantages of exercise on M2 macrophage polarization and energy homeostasis.^41^ We then established an in vivo running model using wild‐type mice and showed that exercise increases bone mass.^42^, ^43^ However, little is known about how macrophages contribute to the mechanical force‐induced osteogenesis of BMSCs. It has been determined that a crucial second messenger for macrophage polarization is Ca^2+^ inward influx mediated by macrophage‐sensitive ion channels.^44^ TGF‐β1 secretion was induced by mechanical stress and may have been triggered by calcium influx into the cytoplasm brought on by Piezo1 activation. Therefore, we knocked down Piezo1 and blocked TGF‐β1 from attaching to its receptor, and surprisingly, both conditions eliminated the rise in bone mass that exercise would have in mice. The recruitment of macrophages to the surface of bone trabeculae is impacted by Piezo1 downregulation, which further affects TGF‐β1 secretion. Our cytological experiments revealed that the polarization of macrophage was time‐dependent, and potentially more accurate assays are required in the future to investigate the function of Piezo1 and TGF‐β1 in bone remodelling.

Although the current work confirmed that Piezo1 mediates mechanical signalling in macrophages in vitro and in vivo, numerous issues remain. Actually, detected TGF‐β1 may also originate from MSCs.^45^ In the future, we may generate macrophage‐specific TGF‐β1 knockout mice which could make our results more persuasive. GsMTx4 and siRNA were used to silence Piezo1, limiting macrophage polarization under mechanical stretch. However, GsMTx4 is not a specific inhibitor of Piezo1.^46^, ^47^ BMSCs and osteoblasts are both able to feel mechanical pressures in vivo, which allows them to influence their own osteogenic development^48^, ^49^ For instance, targeted deletion of Piezo1 in osteoblasts or osteoclasts results in severe osteoporosis.^50^, ^51^ In the future, we may be able to strengthen our ideas by using macrophage‐specific Piezo1 knockout mice in a running model.

## 5 CONCLUSION

**FIGURE 8:**
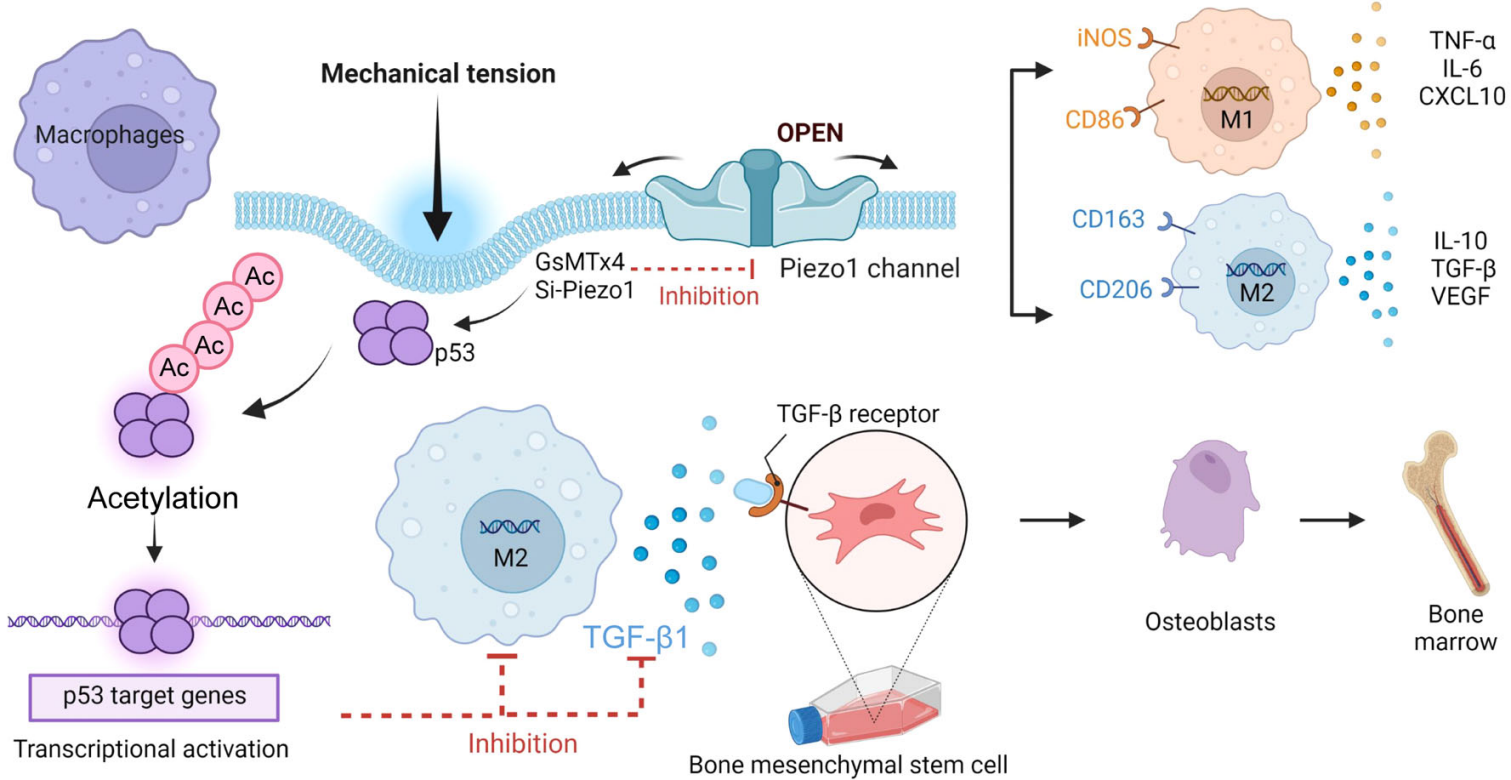
Mechanism diagram. Piezo1‐mediated mechanical tension stimulates macrophage polarization and secretion of TGF‐β1, promoting osteogenic differentiation. Acetylation and deacetylation of p53 play a major role in this process.

In conclusion, we found that mechanical forces mediated changes in calcium inward flow, p53 acetylation and deacetylation through Piezo1, inducing macrophages to polarize towards M2, secreting TGF‐β1 and promoting BMSCs osteogenesis (Figure 8). This demonstrates that Piezo1‐mediated mechanical signalling of macrophages is the basis of osteoimmunology. It provides a clue for future clinical bone trauma, osteoporosis and bone jaw distraction.

## AUTHOR CONTRIBUTIONS

*Conceived and designed the experiments*: Hua Wang and Wei‐Bing Zhang. *Performed the experiments*: Guanhui Cai, Yahui Lu, Weijie Zhong, Ting Wang, Yingyi Li and Xiaolei Ruan. *Analysed the data*: Hongyu Chen, Lian Sun, Zhaolan Guan, Gen Li, Hengwei Zhang, Wen Sun and Minglong Chen. *Wrote the paper*: Guanhui Cai and Yahui Lu.

## CONFLICT OF INTEREST STATEMENT

The authors declare no conflict of interest.

## Supporting information


**Figure S1.** Morphology of macrophages before and after mechanical stretching. (A) Phalloidin staining (Red) of RAW264.7 cells under the tension of 0–6 h. Blue indicates DAPI staining of nuclei. Scale bar, 10 μm.

**Figure S2.** Effect of different concentrations of conditioned medium on osteogenesis of BMSCs. (A) After 7 days, bone marrow mesenchymal stem cells treated with conditioned mediums of macrophages under 2 h of tension were induced into osteogenesis in different concentrations of osteogenic differentiation medium. ALP expression in BMSCs was measured by the ALP staining method. The top are gross scanning images (scale bar: 1 mm), and the lower are enlarged images (magnification: ×250, scale bar: 160 μm). (B) Cell Counting Kit 8 (CCK8) was performed to explore BMSCs proliferation following treatment with different concentrations of conditioned medium.

**Figure S3.** Expression changes of Piezo1 and calcium influx under mechanical tension. (A) The intensity of Ca^2+^ fluorescence marked by Fluo‐3 AM probe under fluorescence microscope after 0–6 h of tension. (B) Immunofluorescent staining of Piezo1 (red) in RAW264.7 cells under the tension of 0–6 h. Blue indicates DAPI staining of nuclei. Three random fields from each time period of the slides of cells were examined. Scale bar, 10 μm. The Piezo1^+^ area were qualified as area values of overlapping fields. (C) mRNA expression of Piezo1 in RAW264.7 cells after tension.

**Figure S4.** Drug stimulation of Yoda1. Piezo1 in RAW264.7 cells with different concentrations of Yoda1‐administration were measured by real‐time RT‐PCR. GAPDH was used for normalization. Data are presented as three biological replicates from three independent experiments.

**Table S1.** List of antibodies used in this research, along with their dilutions and suppliers.
**Table S2.** List of primers used for this study.

## Data Availability

Data are openly available in a public repository that issues datasets with DOIs.
