# Supplementary material for: Piezo1‐mediated M2 macrophage mechanotransduction enhances bone formation through secretion and activation of transforming growth factor‐β1
Source: Cell Prolif. 2023 Mar 7;56(9):e13440. doi: 10.1111/cpr.13440 (PMC10472522; doi:10.1111/cpr.13440)
Supplement: Supplementary file 5 — Table S1. List of antibodies used in this research, along with their dilutions and suppliers. Table S2. List of primers used for this study. [file CPR-56-e13440-s004.docx]

**Supplementary Tables:**

**Supplementary table 1: List of antibodies used in this research, along with their dilutions and suppliers.**

| **Antibody** | **Dilution(Application)** | **Vendor(Cat.#)** |
| --- | --- | --- |
| iNOS | 1:1000(WB) | Abcam(ab178945) |
| Arg-1 | 1:1000(WB) | Abcam(ab124917) |
| β- actin | 1:8000(WB) | Proteintech(66009-1-lg) |
| F4/80 | 1:200(IF) | CST(30325) |
| CD206 AF488 | 0.5mb/ml(FC) | eBioscience(53-2061-80) |
| CD206(M) | 1:10000(IHC) | Proteintech(60143-1-Ig) |
| CD206(R) | 1:200(IF) | Proteintech(18704-1-AP) |
| Col1 | 1:1000(WB) | Millipore(234167) |
| Runx2 | 1:1000(WB) | CST(12556) |
| Osx | 1:1000(WB);1:400(IHC) | Abcam(ab22552) |
| Opn | 1:1000(WB) | Abcam(ab63856) |
| p53 | 1:1000(WB) | CST(2524T) |
| Acetyl-p53 | 1:1000(WB);1:100(IF) | Abcam(ab183544) |
| TGF-β1 | 1:500(IHC) | Abcam(ab215715) |
| Piezo1 | 1:200(IF) | Novus(NBP1-78537) |
| Gapdh | 1:8000(WB) | Beyotime(AG019) |

**Supplementary table 2: List of primers used for this study.**

| **Gene Name** | **Forward Primer(5’-3’)** | **Reverse Primer(5’-3’)** |
| --- | --- | --- |
| *iNOS* | CAGCTGGGCTGTACAAACCTT | CATTGGAAGTGAAGCGTTTCG |
| *Arg-1* | AACACTCCCCTGACAACCA | CATCACCTTGCCAATCCC |
| *MRC1* | GGTGCTACTCCGAACAACAG | ACCGTGGCTGAAAGTTCCT |
| *IL-10* | CCTGGATCTGTATCACCGAAGC | CTCCGACCACTCTGCCTTGTTA |
| *Ocn* | CTGACCTCACAGATGCCAAGC | TGGTCTGATAGCTCGTCACAAG |
| *Col1* | GCTCCTCTTAGGGGCCACT | CCACGTCTCACCATTGGGG |
| *Osx* | TGAAAGGTCAGCGTATGGCTT | ATGGCGTCCTCTCTGCTTG |
| *Opn* | AGCAAGAAACTCTTCCAAGCAA | GTGAGATTCGTCAGATTCATCCG |
| *Runx2* | GACTGTGGTTACCGTCATGGC | ACTTGGTTTTTCATAACAGCGGA |
| *Alp* | TCCTGACCAAAAACCTCAAAGG | TGCTTCATGCAGAGCCTGC |
| *TGF-β1* | GCAACAATTCCTGGCGTTACC | CAGTGAGCGCTGAATCGAAAG |
| *Piezo1* | TCCTGCTGTATGGGCTGAC | GGTAGCGTGTGTGTTCCAGTC |
| *Hvcn1* | CATCTGACCCAACACCACAG | CAGCTTCTTCTTCCCGTTTG |
| *Trpm7* | CCTCATGAAGACCATTTTCTAA | ACAACTGTAACCTTCCTCACAG |
| *Trpa1* | GTCCAGGGCGTTGTCTATCG | CGTGATGCAGAGGACAGAGAT |
| *Trpc6* | GCAGGATTTCGTTGTTGGT | TGCTGACAGTTGGATGAGC |
| *Piezo2* | CTCACCTTTCCTGGCGTCAT | CCTCTTGAAACTCAGGCAGT |
| *Trpc4* | TCACCTTCGTGCTCCTGTTG | AGATGTGCTTGCTCTCCTTG |
| *Trpc1* | GCCATCTTTGTCACCAGGTT | GCTCGAGCAAACTTCCATTC |
| *Trek1* | ATTGTGCATGGTGACCTCAA | TGCTGACAGTTTGGATGAGC |
| *Kcnk10* | TGGCTGCATCGTGTTTGTGA | CTGTGGTCAGCGTGACTACC |
| *Ptgs2* | TTCCAATCCATGTCAAAACCGT | AGTCCGGGTACAGTCACACTT |
| *Socs3* | TGCGCCTCAAGACCTTCAG | GCTCCAGTAGAATCCGCTCTC |
| *Cxcl10* | CCAAGTGCTGCCGTCATTTTC | GGCTCGCAGGGATGATTTCAA |
| *Edn1* | TTTCCCGTGATCTTCTCTCTGC | CTGAGTTCGGCTCCCAAGAC |
| *Gapdh* | AGGTCGGTGTGAACGGATTTG | TGTAGACCATGTAGTTGAGGTC |

**Supplementary Figure legends**

**Supplementary Figure 1: Morphology of macrophages before and after mechanical stretching.**

(A)Phalloidin staining (Red) of RAW264.7 cells under the tension of 0-6 hours. Blue indicates DAPI staining of nuclei. Scale bar, 10μm.

**Supplementary Figure 2: Effect of different concentrations of conditioned medium on osteogenesis of BMSCs.**

(A)After 7 days, bone marrow mesenchymal stem cells treated with conditioned mediums of macrophages under 2 hours of tension were induced into osteogenesis in different concentrations of osteogenic differentiation medium. ALP expression in BMSCs was measured by the ALP staining method. The top are gross scanning images (scale bar: 1 mm), and the lower are enlarged images (magnification: × 250, scale bar: 160μm).

(B) Cell Counting Kit 8 (CCK8) was performed to explore BMSCs proliferation following treatment with different concentrations of conditioned medium.

**Supplementary Figure 3: Expression changes of Piezo1 and calcium influx under mechanical tension.**

(A)The intensity of Ca^2+^ fluorescence marked by Fluo-3AM probe under fluorescence microscope after 0-6 hours of tension.

(B)Immunofluorescent staining of Piezo1 (red) in RAW264.7 cells under the tension of 0-6 hours. Blue indicates DAPI staining of nuclei. Three random fields from each time period of the slides of cells were examined. Scale bar, 10μm.The Piezo1^+^ area were qualified as area values of overlapping fields.

(C)mRNA expression of Piezo1 in RAW264.7 cells after tension.

**Supplementary Figure 4: Drug stimulation of Yoda1.**

Piezo1 in RAW264.7 cells with different concentrations of Yoda1-administration were measured by real-time RT-PCR. GAPDH was used for normalization. Data are presented as three biological replicates from three independent experiments.
